# The Nutritional Value, Biochemical Traits, and Growth of *Brassica oleracea* Grown Under Red, Blue, and Combined Red–Blue LED Lighting

**DOI:** 10.3390/plants14233700

**Published:** 2025-12-04

**Authors:** Maryam Haghighi, Mohammad Reza Moradian, Maryam Mozafarian, András Geösel

**Affiliations:** 1 Department of Horticulture, College of Agriculture, Isfahan University of Technology, Isfahan 84156-83111, Iran; 2 Department of Vegetable and Mushroom Growing, Hungarian University of Agriculture and Life Sciences, 1118 Budapest, Hungary

**Keywords:** antioxidant activity, glucosinolates, *Brassica oleracea*, growth parameters, nutrition quality, supplementary light

## Abstract

Brassica vegetables are in high demand because they are an essential nutrient source for humans. Glucosinolates (GSLs), a major bioactive compound found in Brassicaceae, are amino acid derivatives that contribute to the health benefits of these crops. Light quality plays a significant role in plant growth and metabolite synthesis, and light-emitting diodes (LEDs) as artificial light sources offer many benefits. This study examined three cultivars of leafy cabbage *B. oleracea.* var. acephala (Kale), *B. oleracea* var. viridis (collard), and *B. oleracea* var. capitata (cabbage) grown under different LED conditions (red, blue, and blue–red) in the growing chamber. The primary objective of this study was to identify the most effective LED light spectrum for promoting GSLs accumulation and enhancing the overall plant quality. The findings of this study demonstrate that LED lights can have varying impacts on the cultivars of leafy cabbage. The different light spectra had varying impacts on the parameters examined in this study. GSLs compounds, particularly glucobrassicin, showed the most significant increase under the blue light treatment, with a 61% increase compared to the control. The R&B (red and blue) light treatment was the most effective in improving the growth traits of the shoot and root in the Kale cultivar. For the collard cultivar, the R&B light increased the leaf length and width, whereas for the cabbage cultivar, it led to an increase in the number of leaves and chlorophyll index. These findings demonstrate that the specific light quality can have different effects on the phytochemical composition and morphological characteristics of the different leafy cabbage cultivars. The blue light spectrum was particularly effective in enhancing GSLs accumulation, while the combination of red and blue light provided the most beneficial effects on overall plant growth and development across the three cultivars studied. These results suggest that the metabolism and phytochemical properties of leafy cabbage cultivars depend on exposure to multiple factors, such as cultivar type and light quality. Therefore, R&B light was the most effective light for most traits and can be suggested for performance.

## 1. Introduction

Brassica vegetables are in high demand because they are an essential nutrient source for humans [1]. Brassica vegetables such as Chinese cabbage (*Brassica rapa* L. ssp. pekinensis), kale (*Brassica oleracea* var. acephala), broccoli (*Brassica oleracea* var. italica), and cauliflower [2] are rich in minerals, vitamins, and phytochemicals, including glucosinolates (GSLs), phenolic, carotenoids, and anthocyanin compounds [3]. Sulforaphane and other GSLs hydrolysis products have notable anticancer properties [4]. GSLs, a major bioactive compound found in *Brassicaceae*, is an amino acid derivative known for its antioxidant and cancer-protective activities [5]. Recent research has mapped GSL profiles in a variety of Chinese cabbage [2], showing that they are influenced by genotype and growth environment, including light, temperature, fertilizer, and biotic and abiotic damage [6]. GSLs are phytochemicals that are proposed to be an important contributor to the health benefits of these vegetables [7].

Light quality, intensity, and duration play significant roles in plant growth and metabolite synthesis, prompting scientists and farmers to experiment with different light sources. Light is one of the most crucial factors in plant growth and metabolite synthesis. It is possible that the qualities of light, such as its quality and intensity, as well as the duration of daylight and the day-to-night cycle, will play a significant role in its exploration [8]. Because each type of light source emits a distinct wavelength, scientists and farmers have been encouraged to switch and combine their light sources. In greenhouses, artificial light is primarily responsible for vegetative growth, which is required for plants to produce flowers or fruit [9].

Light-emitting diodes (LEDs) as artificial light have many benefits, including a long lifespan, the ability to produce light of a specific wavelength, minimal energy waste, and the ability to control the amount of light emitted by the device. Experiments were conducted with cucumber, lettuce, and tomato seedlings using various red light to blue light ratios (Red: Blue) [10]. All metabolites in specialized crops are elevated under LED lighting because the plants only receive blue and red wavelengths, as opposed to the other wavelengths present in the spectrum of fluorescent/incandescent bulbs [11]. The examples in Table 1 demonstrate the positive effects of the LED lighting sources and ratios on various vegetables.

The amount and quality of light influence numerous plant traits, including plant height, internode length, branch number, and leaf size [11]. When there is an increase in energy at the leaf surface, the transpiration rate may occasionally decrease. This, in turn, increases the temperature at the plant shoot, which inhibits plant growth. LEDs provide less infrared light than sunlight and other lighting sources, resulting in a cooler plant canopy [18].

LED light can also affect the synthesis of secondary metabolites [19,20], chlorophyll [21], endogenous abscisic acid (ABA) [18,20,22], and antioxidant activity [23]. Among these, the xanthophyll cycle, re-oxidation of lowered balances by photosynthesis, malate pathway, and antioxidant activity [20]. Irradiating Chinese cabbage seedlings with blue LED light increased ascorbic acid levels and decreased the number of reactive oxygen species (ROS) [1]. Important dietary components, such as glucoraphanin, glucobrassicin, polyphenols, and anthocyanins, can be increased using red LED lights while cultivating Chinese cabbage and *B. oleracea.* var. acephala [2]. In a study by Guo et al. [24], green LED irradiation promoted GSLs profile accumulation, including glucoraphanin, glucoiberin, gluconapin, gluconasturtiin, glucobrassicin, and sinigrin in cabbage.

The application of either individual or combined blue, red, or white LEDs influences the expression of key regulatory genes involved in carotenoid and flavonoid biosynthesis in wheat sprouts [25] and affects the antioxidant defense systems in post-harvest broccoli [26]. LED lighting also promotes the accumulation of phenylpropanoids, tocopherols, vitamin C, and ascorbate [27]. The activity and gene expression of enzymatic antioxidants, such as catalase and glutathione S-transferase, may also be influenced [28]. In *B. oleracea.* var. acephala, the levels of health-promoting compounds such as GSLs, anthocyanins, flavonoids such as kaempferol and quercetin, phenolic compounds, and glutathione were evaluated in both flower stalks and leaves, with the flower stalks showing higher concentrations of these beneficial compounds compared with the leaves [16].

Previous studies have demonstrated that various combinations of LED light spectra can significantly influence plant growth and metabolite synthesis, with the outcomes differing according to the plant species and environmental conditions. However, there is a critical gap in research that specifically examines the effects of these light spectra on GSL compounds in Brassica vegetables, particularly in leafy cabbages such as *B. oleracea.* var. acephala, *B. oleracea* var. viridis, and *B. oleracea* var. capitata. These cultivars are not only popular in diets worldwide but are also rich in the bioactive compound GSLs. Despite their importance, the synthesis of such compounds in response to varying light conditions remains underexplored. This study hypothesizes that the application of red, blue, and blue-red LED light will enhance the growth, physiological traits, and nutritional properties of these cabbage varieties, particularly by increasing the GSLs content compared with the standard control light. The primary objective of this study was to determine which of these LED light spectra is most effective at promoting GSLs accumulation and improving the overall plant quality. By evaluating the impact of these light treatments on key growth parameters, analyzing biochemical properties, including GSLs levels, and assessing the mineral composition of plants, this research aims to bridge the existing knowledge gap regarding the influence of environmental conditions on GSLs accumulation. Ultimately, by identifying the optimal light conditions for enhancing the nutritional value of *B. oleracea.* var. acephala (Kale), *B. oleracea* var. viridis (collard) and *B. oleracea* var. capitata (cabbage), this study aims to contribute to improved dietary options and health benefits for consumers.

## 2. Results

### 2.1. Effect of LED Spectra on the Morphological Characteristics of the Three Types of Leafy Cabbage

Based on the results from the analysis of variance, the main effect of variety did not show a significant effect on the fresh and dry weight of shoots and roots, as well as leaf length. However, there were significant effects at the 1% probability level for petiole length, shoot length, width, and leaf number. The main effect of light did not have a significant effect on the fresh weight of the roots, but it did have a significant impact on other morphological traits at the 1% probability level. The interaction between variety and light had a significant effect on all the morphological traits of cabbage at the 1% probability level (Table S1 Supplementary File). The main effects of the treatments on the morphological characteristics are presented in Supplementary Table S2.

**Figure 1:**
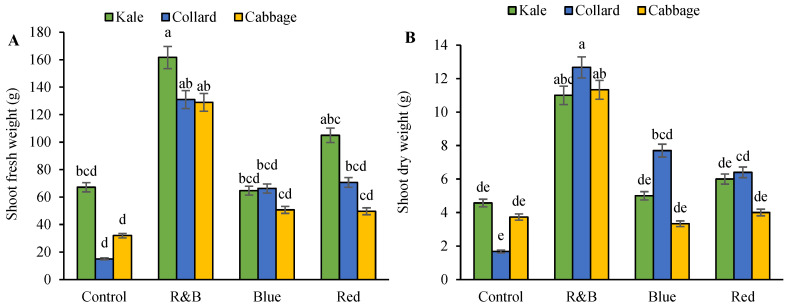

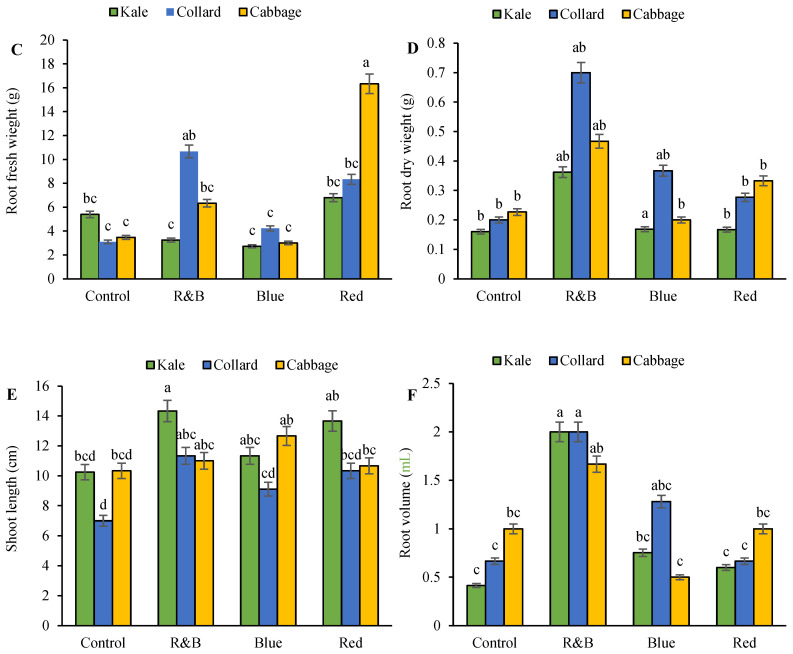
The interaction between LEDs on three leafy cabbages on shoot fresh weight (**A**), shoot dry weight (**B**), root fresh weight (**C**), root dry weight (**D**), shoot length (**E**), and root volume (**F**). According to the least significant difference test, significant differences between treatments are shown by various letters at *p* < 0.05. Each bar indicates the mean value standard deviation. Different letters indicate statistically significant differences between the treatment groups; for example, if two bars have different letters, it signifies a significant difference, while bars with the same letter indicate no significant difference.

The fresh weight of the shoots increased when we used the red-blue combination lights. The lowest levels of this trait were observed in the collards and cabbage under the control and blue and red light conditions. Kale also exhibited low shoot fresh weight under control and blue light conditions (Figure 1A). Shoot dry weight increased significantly in response to R&B treatment for all cultivars and all LED conditions for the collards (Figure 1B). The highest root fresh weights were observed in the cabbage and collard varieties under red and R&B light conditions, respectively. All varieties under the control and blue light conditions had the lowest root fresh weight (Figure 1C). The highest root dry weight was observed in kale and collard varieties under blue light and all varieties under R&B light, whereas all varieties under control and red light had the lowest root dry weight (Figure 1D). Overall, the shoot length in all varieties was greater under different light spectra compared to the control. The kale variety exhibited the longest shoot length under R&B light, followed by red and blue light. In contrast, the cabbage variety showed increased shoot length under blue and R&B light compared with the other light intensities (Figure 1E). Across all three cultivars, root volume was greatest under R&B light and lowest under control and red light conditions (Figure 1F).

**Figure 2:**
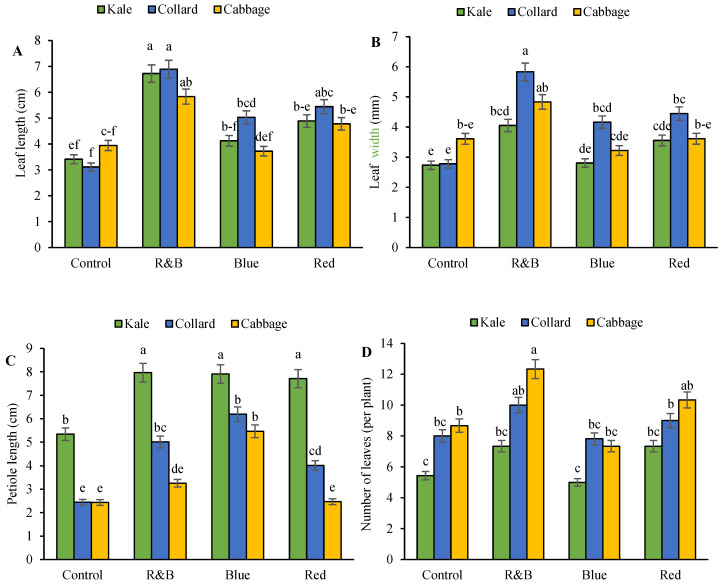
The interaction between the LEDs on three leafy cabbages on leaf length (**A**), leaf width (**B**), petiole length (**C**), and the number of leaves (**D**). According to the least significant difference test, significant differences between treatments are shown by various letters at *p* < 0.05. Each bar indicates the mean value standard deviation. Different letters indicate statistically significant differences between the treatment groups; for example, if two bars have different letters, it signifies a significant difference, while bars with the same letter indicate no significant difference.

The longest leaf length was observed in all three cultivars under R&B light, whereas the shortest leaf length was observed under control light in all three cultivars (Figure 2A). The largest leaf width was also observed in all three cultivars under R&B light (Figure 2B). The petiole length was greater in the kale cultivar than in the other two cultivars and increased under blue, red, and R&B light spectra compared with the control. The shortest petiole length was observed in the cabbage under control and red light (Figure 2C). Additionally, the number of leaves per plant was higher in all three varieties under R&B light than under the other light spectra. Notably, kale had the shortest shoot length under all light conditions compared to the other two varieties (Figure 2D).

### 2.2. Effects of LED Spectra on the Physiological Characteristics of the Three Leafy Cabbage Varieties

**Figure 3:**
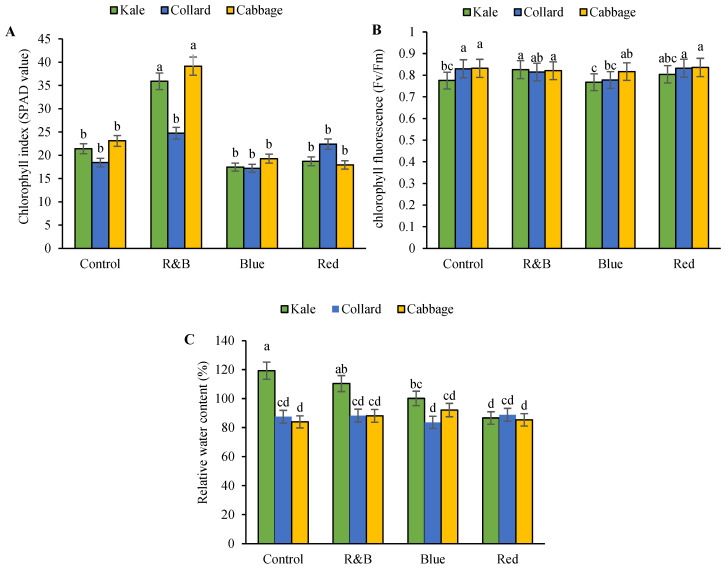
The interaction between the LED on three leafy cabbages on the chlorophyll index (**A**), chlorophyll fluorescence measurements (**B**) and relative water content (**C**). According to the least significant difference test, significant differences between treatments are shown by various letters at *p* < 0.05. Each bar indicates the mean value standard deviation. Different letters indicate statistically significant differences between the treatment groups; for example, if two bars have different letters, it signifies a significant difference, while bars with the same letter indicate no significant difference.

According to the results from the analysis of variance, the main effect of variety exhibited a significant effect at the 1% probability level on the chlorophyll fluorescence and relative water content of the leaves, whereas it did not significantly affect the chlorophyll index. Additionally, light significantly affected the chlorophyll index, chlorophyll fluorescence, and relative water content at the 1% probability level. The interaction between variety and light significantly influenced the chlorophyll index, chlorophyll fluorescence, and relative water content at the 1% probability level (Table S3 Supplementary File). The main effects of the treatments on the physiological characteristics are presented in Supplementary Table S4. The chlorophyll index was higher in the kale and cabbage cultivars under R&B light than in the other light conditions (Figure 3A).

In the cabbage cultivar, chlorophyll fluorescence did not show significant differences among the various light spectra, whereas in the kale and collard cultivars, the lowest chlorophyll fluorescence was observed under blue light and the highest under R&B light (Figure 3B). Overall, the relative water content was higher in the kale cultivar than in the other two cultivars, and the highest levels were observed under the control and R&B light conditions compared with the other light spectra. In contrast, the relative water content in the collard and cabbage did not differ significantly among the light intensities (Figure 3C).

### 2.3. Effect of LED Spectra on the ABA Content and Antioxidant Enzyme Activity of the Three Leafy Cabbage Varieties

**Figure 4:**
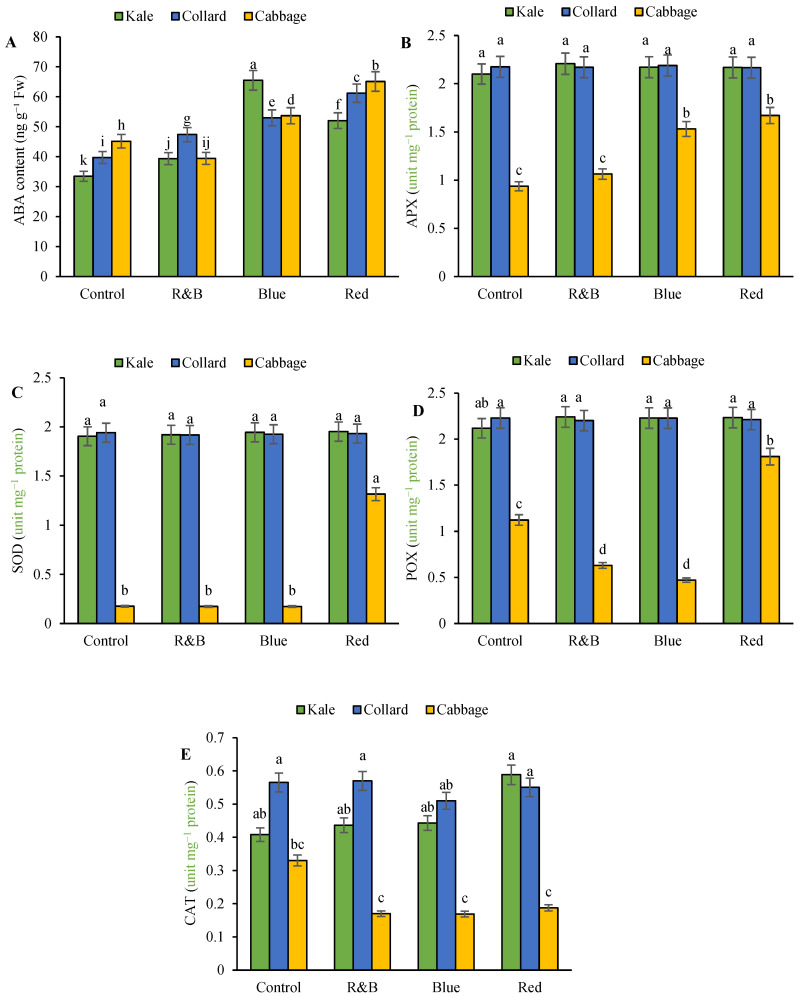
The interaction between LED on three leafy cabbage on ABA concentration (**A**), ascorbate peroxidase (**B**), superoxide dismutase (**C**), peroxidase (**D**), and catalase (**E**) According to the least significant difference test, significant differences between treatments are shown by various letters at *p* < 0.05. Each bar indicates the mean value standard deviation. Different letters indicate statistically significant differences between the treatment groups; for example, if two bars have different letters, it signifies a significant difference, while bars with the same letter indicate no significant difference.

The analysis of variance results indicated that the main effect of variety had a significant impact at the 1% probability level on ABA, APX, SOD, POX, and CAT. Light did not have a significant effect on ABA, SOD, and CAT, but it did have a significant effect at the 1% probability level for APX and POX. The interaction between variety and light significantly affected ABA, APX, SOD, POX, and CAT at the 1% probability level (Table S3 Supplementary File). The main effects of the treatments on the enzyme activity are presented in Supplementary Table S4. The highest ABA was observed in all varieties under blue and red light, whereas the lowest ABA was observed in the kale variety under control light (Figure 4A). Overall, the activities of the antioxidant enzymes APX, SOD, and POX were higher in the kale and collard cultivars than in cabbage, and there were no significant differences in the activity of these enzymes among the different light spectra in kale and collard. The lowest activities of these enzymes were observed in cabbage under control light (Figure 4B–D). The catalase (CAT) enzyme activity was also specifically higher in kale and collard cultivars than in cabbage. In the cabbage cultivar, the highest level of CAT was observed under the control light, and there were no significant differences among the other light spectra (Figure 4E).

### 2.4. Effect of LED Spectra on the GSLs Content of the Three Leafy Cabbage Varieties

**Figure 5:**
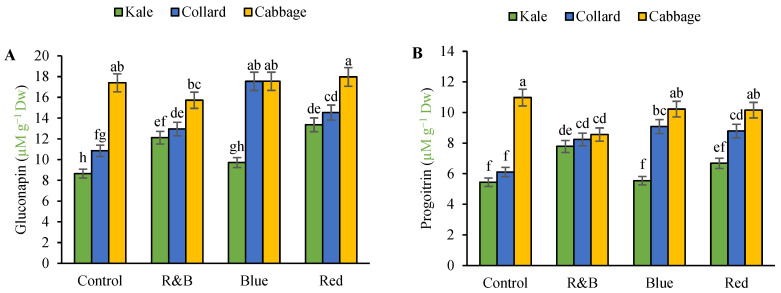

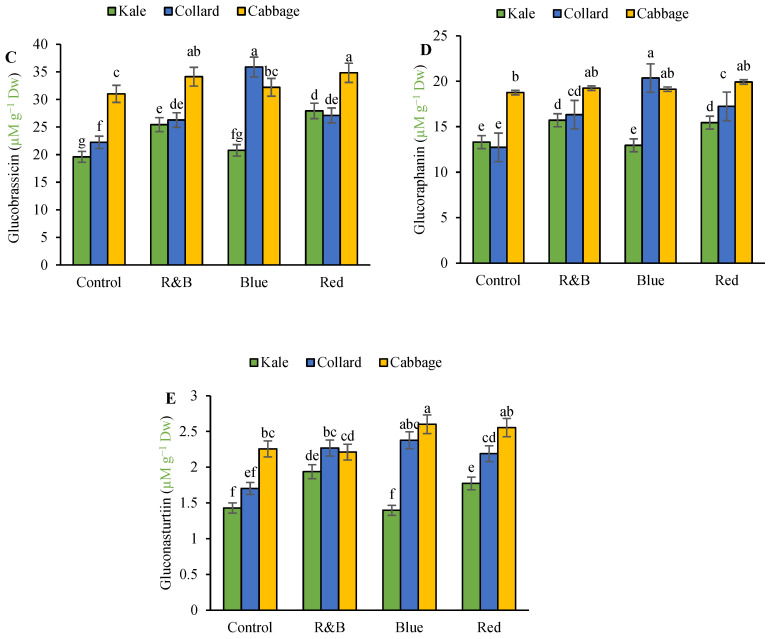
The interaction between LED on three leafy cabbage on gluconapin (**A**), progoitrin (**B**), glucobrassicin (**C**), glucoraphanin (**D**), and gluconasturtiin (**E**). According to the least significant difference test, significant differences between treatments are shown by various letters at *p* < 0.05. Each bar indicates the mean value standard deviation. Different letters indicate statistically significant differences between the treatment groups; for example, if two bars have different letters, it signifies a significant difference, while bars with the same letter indicate no significant difference.

Furthermore, the results showed that the main effect of variety significantly influenced progoitrin, glucolipin, glucobrassicin, gluconasturtiin, and glucoraphanin at the 1% level. Moreover, the main effect of light significantly influenced all GSL compounds at the 1% probability level. The interaction between varieties had a significant impact on all GSL compounds at the 1% probability level. (Table S5 Supplementary File). The main effects of the treatments on the GSLs content are presented in Supplementary Table S6. In general, the highest levels of GSLs were observed in cabbage and the lowest levels were observed in kale. Gluconapin levels in cabbage increased under red, blue, and control light conditions compared with R&B light, whereas in the collard cultivar, the highest gluconapin was observed under blue light compared with the other light spectra. The lowest glucolipin levels were observed under the control light and in the kale cultivar (Figure 5A). The highest levels of progoitrin were observed in the cabbage cultivars under the control light compared with the R&B light, whereas no significant difference was observed under the blue and red light. The lowest progoitrin levels were observed under the control and blue light conditions in the kale cultivar (Figure 5B). The highest level of glucobrassicin was observed in the collard and cabbage cultivars under blue and red light, respectively, and the lowest level was observed in both cultivars under control light (Figure 5C). In both the collard and cabbage cultivars, the highest level of glucoraphanin was observed under blue light compared with the control, and the lowest level of glucoraphanin was observed in the kale cultivar under control and blue light (Figure 5D). The highest level of gluconasturtiin was observed in both cabbage and collard cultivars under blue light, whereas the lowest level was observed in both cultivars under control light (Figure 5E).

### 2.5. Effect of LED Spectra on the Mineral Content of the Three Types of Leafy Cabbage

**Figure 6:**
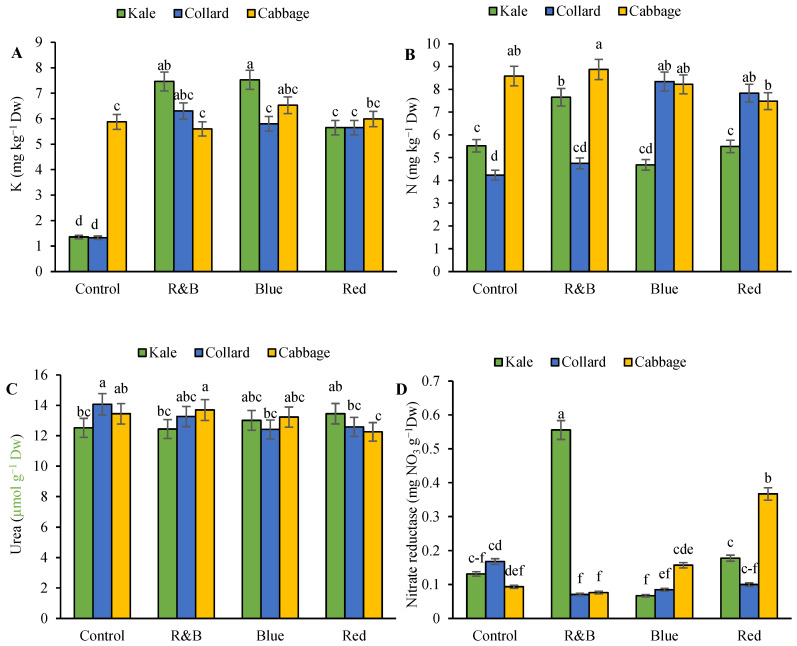
The interaction between LED on three leafy cabbage on K (**A**), N (**B**), Urea (**C**), and nitrate reductase (**D**). According to the least significant difference test, significant differences between treatments are shown by various letters at *p* < 0.05. Each bar indicates the mean value standard deviation. Different letters indicate statistically significant differences between the treatment groups; for example, if two bars have different letters, it signifies a significant difference, while bars with the same letter indicate no significant difference.

Lastly, the analysis revealed that the main effect of variety had a significant effect at the 1% probability level on potassium, nitrogen, and nitrate reductase, whereas there was no significant difference regarding urea. Lastly, the main effect of light had a significant effect at the 1% probability level on potassium, nitrogen, and nitrate reductase, whereas there was no significant difference observed for urea. The interaction between variety and light significantly influenced potassium, urea, nitrogen, and nitrate reductase at the 1% probability level (Table S5 Supplementary File). The main effects of the treatments on the mineral content are presented in Supplementary Table S6. The lowest potassium values were measured without artificial lighting in kale and collard. All light treatments significantly increased the potassium concentrations in kale and collard. There were no significant differences between the control and light-treated cabbage in terms of potassium levels (Figure 6A). According to the findings, the nitrogen concentration of kale and collard significantly increased under R&B and blue light conditions compared to the control (Figure 6B).

The collar and cabbage varieties had the highest urea under the control and R&B light conditions, whereas the kale varieties had low urea under the same light conditions. Kale had high urea under blue and red light, whereas collard and cabbage had low urea under red light (Figure 6C). Specifically, nitrate reductase activity was increased in the kale and cabbage cultivars under R&B and red light, respectively, compared with the other light intensities (Figure 6D).

### 2.6. Principal Component Analysis of All Measured Parameters Treated with LED on Three Leafy Cabbage

**Figure 7:**
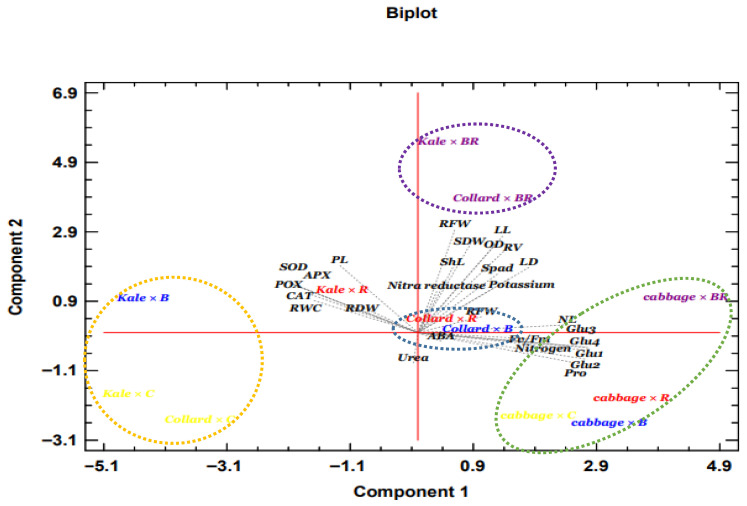
Principal component analysis of the interaction between LED and the three leafy cabbages in all of the observed parameters. Control light (C), blue light (B), red light (R), and blue-red light (BR). PL: petiole length, ShL: shoot length, OD: opening diameter, LD: leaf width, NL: number of leaves per plant, LL: leaf length, Spad: chlorophyll index, RV: root volume, Fv/Fm: chlorophyll fluorescence measurements, RFW: root fresh weight, SDW: shoot dry weight, RFW: root fresh weight, RDW: root dry weight, RWC: relative water content, ABA: ABA, APX: ascorbate peroxidase, SOD: superoxide dismutase, POX: peroxidases, CAT: catalase, Glu1: gluconapin, Pro: progoitrin, Glu2: gluconapin, Glu3: glucobrassicin, Glu4: gluconasturtiin.

Principal component analysis (PCA) was used to determine the most significant global impacts of LED light quality on the growth, phytochemical composition, and mineral elements of the three cabbage cultivars (Figure 7). The PCA results indicated that the nitrogen and potassium concentrations in the red and blue treatments were favorably correlated with the fresh weight and chlorophyll contents (Kale × R&B and Collard × R&B). This correlation may be attributed to the enhanced light absorption and use under these wavelengths, which promotes photosynthesis and nutrient uptake by the plants. The most significant changes were observed under R&B light in terms of growth traits and nitrogen metabolism, particularly the activity of nitrate reductase, which was reflected in the changes in the potassium nitrate levels in Collard and Kale. These findings are represented in a distinct category (purple circle) in the figure. The differential responses observed among the cultivars could be linked to their unique genetic and morphological characteristics, which may influence the efficiency of light harvesting, photosynthetic capacity, and nutrient assimilation pathways. Biochemical changes, particularly in GSLs, were most pronounced under R&B light in cabbage, while ABA levels showed the greatest variation when red or blue light was used alone in Collard. Additionally, there was a strong correlation between GSL levels and cabbage cultivars (Cabbage × Control, Cabbage × R&B, Cabbage × Blue, Cabbage × Red). This suggests that specific cultivars may have varying capacities for synthesizing GSLs in response to different light qualities, possibly due to genetic factors influencing the expression and regulation of the enzymes and transcription factors involved in the GSL biosynthesis pathways. The observed differences in GSLs accumulation among the cultivars could also be related to their distinct morphological traits, such as leaf structure and surface area, which may affect the light absorption and signaling processes that modulate secondary metabolite production. In Kale × Control, Kale × R&B, and Collard × Control treatments, the highest antioxidant activity was recorded under both natural and red light, with the vegetative traits and secondary metabolites showing significant divergence. This divergence may indicate that the light spectrum influences the production of secondary metabolites and affects overall plant growth. Notably, the least variation in antioxidant enzyme activities was observed under red light when using Collard × Control and Kale × Control, which showed the furthest distance from the measured traits in the PCA. Similarly, minor changes were noted in Cabbage × Control, Cabbage × Red, and Cabbage × Blue treatments when monochromatic lights were applied. The most significant positive changes were observed when the blue and red lights were used simultaneously, especially in Collard, Kale, and Cabbage, indicating a synergistic effect. Growth traits, including root fresh weight, shoot dry weight, opening diameter, leaf length, shoot length, and leaf width, showed a greater correlation with each other in the upper quadrant of the PCA plot. Interestingly, the activity of nitrate reductase exhibited a negative correlation with urea levels, suggesting that as nitrate reductase activity increased, urea levels decreased under R&B light, indicating enhanced nitrogen metabolism. Furthermore, the changes in the shoot traits were more pronounced under R&B light compared with the root traits, with the shoot traits located in the right quadrant and the root traits in the left quadrant of the PCA plot. Overall, these findings underscore the intricate interplay between light quality and plant physiological responses, highlighting the potential for optimizing light conditions to enhance the growth and phytochemical profiles of cabbage cultivars.

### 2.7. Spider Diagram

**Figure 8:**
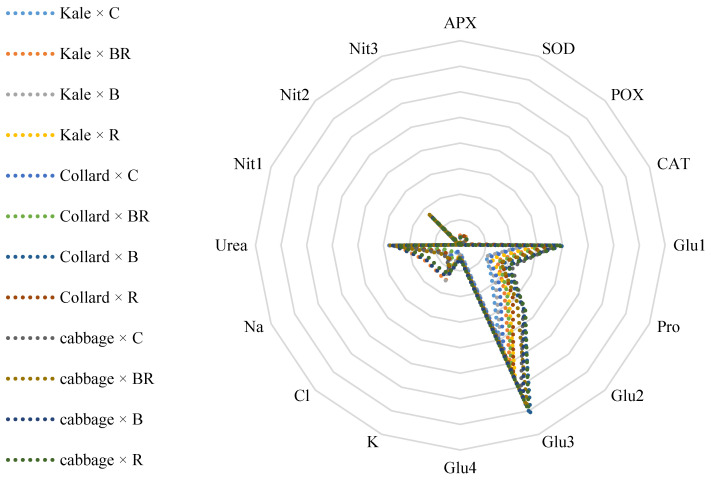
A spider diagram depicting the relationship between the application of LED lighting to three different green cabbages and the nutritional value criteria. Control light (C), blue light (B), red light (R), blue-red light (BR). APX: ascorbate peroxidase, SOD: superoxide dismutase, POX: peroxidases, CAT: catalase, Glu1: glucoraphanin, Pro: progoitrin, Glu2: gluconapin, Glu3: glucobrassicin, Glu4: gluconasturtiin, K: potassium, chlorine (Cl), sodium (Na), Urea: urea, Nit1: nitrate, Nit2: nitrogen, Nit3: nitrate reductase.

The results presented in Figure 8 demonstrate significant changes in glucoraphanin, glucobrassicin, urea, and potassium levels under the influence of the LED light. The most notable changes were observed in glucoraphanin, urea, and nitrogen, with the highest alterations occurring in Collard × B and Cabbage × B. These changes indicate that light affects nitrogen metabolism, leading to variations in nitrogen and urea levels, with potassium playing a crucial role in this context. These findings suggest that specific light wavelengths can enhance the biosynthesis of GSLs, which are known for their health-promoting properties. Previous studies, such as those by Demir et al. [29], have shown that red and blue light spectra promote GSLs accumulation in Brassicaceae plants, which is consistent with our findings. Additionally, the observed low potassium levels may result from altered nutrient uptake efficiency under different light conditions, as LED light can influence root development and nutrient absorption dynamics. While significant biochemical changes were noted in the levels of glucoraphanin and glucobrassicin, the least variation was observed in the activities of the antioxidant enzymes POX, CAT, and APX. This suggests that the quality of light not only affects the phytochemical composition but also plays a crucial role in plant nutrient dynamics.

## 3. Discussion

### 3.1. Effect of the LED Spectra on the Morphological Characteristics

The application of LED lighting has shown significant effects on various members of the Brassica family, including Chinese cabbage [30], *B. oleracea.* var. acephala [31], broccoli [29], and white cabbage [32]. Notably, Demir et al. [29] found that both the fresh and dry weights of the microgreens of broccoli and cabbage increased when exposed to white, red, and red + far-red light conditions, as well as with red + blue + far-red. In a production system, the combination of blue and red LEDs can improve both crop quality and yield [2,33]. Red leaf lettuce seedlings grown under a combination of red and blue light had larger leaves and more fresh weight than those grown under red light alone [34]. Additionally, Zhang et al. [16] reported that the total fresh weight of Chinese kale was significantly increased at a red-to-blue light ratio of 8:1. The results of the present study showed that the shoot fresh and dry weight of kale increased by 140 and 144%, respectively, under R&B light compared with the control light. Red and blue light are particularly effective because they are strongly absorbed by photosynthetic pigments, thereby enhancing photosynthesis and promoting plant growth and development [29].

The effect of red-blue LED light at a ratio of 1:1 on the stem diameter, height, and root growth of tomato (*Solanum lycopersicum*) is consistent with the findings of the present study [35]. The ratio of plant hormones is one mechanism by which light influences the growth rate and morphology of plants. Long-term cultivation under varying light conditions induces homeostasis by altering internal hormone levels, specifically auxin. Blue light appears to alter the ratio of auxin to cytokinin by preventing or destroying auxin production. It has been demonstrated that blue light degrades indole acetic acid. Therefore, when only blue light is used, indole acetic acid is destroyed, and plant growth is stunted and internode development occurs [36].

Although plants grown in high light produce smaller leaves than those grown in low light, the total leaf area of plants grown in high light is frequently greater because they produce more leaves [20]. Plant growth and development are primarily dependent on photosynthesis. The red and blue regions of the spectrum are absorbed by the photosynthetic pigments in plant leaves during photosynthesis [37]. According to a study by He et al. [38], far-red light treatment significantly influenced the morphology of Chinese kale baby leaves, leading to notable increases in leaf area, main stem length, and petiole length. In our current study, the collards exhibited remarkable growth under red and blue light conditions, with the leaf width and length increasing by 108% and 121%, respectively, compared to the control light conditions. The pronounced enhancement in leaf size and overall morphology can be attributed to the specific wavelengths of light used in the LED treatments. Red and blue light are critical for photosynthetic efficiency and can stimulate hormonal responses that promote cell division and elongation [39].

### 3.2. Effect of the LED Spectra on the Physiological Characteristics

A strong correlation exists between the chlorophyll concentration and plant growth [40]. In a study by Frede et al. [41], pak choi (*Brasica rapa* var chinensis) sprouts grown under white LED light exhibited higher chlorophyll content than those cultivated under blue and red LED lights. Conversely, Li et al. [42] reported that choy sum sprouts under a light ratio of 1 red to 2 blue (1R2B) had a greater photosynthetic pigment content than those under a ratio of 2 red to 1 blue (2R1B). Our findings revealed that the chlorophyll content in the kale and cabbage varieties increased by 67.6% and 69.4%, respectively, under R&B light compared with the control group. The blue light spectrum (425–490 nm) and red light spectrum (610–700 nm) are the most effective wavelengths for plant photosynthesis [43]. The varying ratios of blue to red light play a crucial role in optimizing the combination of light to investigate their impact on the content of photosynthetic pigments [44]. Blue light not only enhances the chlorophyll content but also, when combined with red light, triggers a signaling cascade that promotes stomatal opening [45]. This process increases the stomatal conductance, thereby allowing for greater CO_2_ uptake and water loss regulation. Furthermore, the combination of red and blue light enhances photosynthetic activity by providing superior excitation of photoreceptors such as phytochromes, cryptochromes, and phototropin compared with monochromatic red or blue light [45], which increases the chlorophyll content in plants.

The chlorophyll fluorescence ratio serves as a quick probe of the photosynthetic efficiency of photosystem II (PSII) and provides valuable insights into the light energy conversion efficiency, although it does not fully capture the complexities associated with photoinhibition [46]. Optimal ratios of blue and red light intensities significantly enhance the photochemical efficiency of PSII, electron transport, and overall photosynthesis [15]. In a study by Li et al. [47], chlorophyll fluorescence in Chinese cabbage under various LED light spectra did not exhibit significant differences compared with the control group. Our findings indicate that the effects of different light spectra on chlorophyll fluorescence differ among cabbage varieties. While the results for the cabbage variety align with those of Li et al. [47], we observed that in the collard variety, the chlorophyll fluorescence under blue light was lower than that under other light spectra. This discrepancy may be attributed to the unique physiological responses of collards to blue light, which could limit their ability to effectively utilize this spectrum for photosynthesis compared with other varieties.

RWC is a reliable method for assessing the osmotic pressure and evaluating the water balance in plants. Our research revealed cultivar-specific responses to different light spectra. In both the collard and cabbage varieties, no significant differences were observed between the light treatment and control groups. However, in kale, the lowest RWC was recorded under red light compared with the other light spectra, whereas the highest RWC in this cultivar was recorded under control and R&B light conditions. Vitale et al. [48] noted that R&B light treatment resulted in increased leaf thickness and a higher percentage of intercellular spaces, which likely enhanced water movement within the leaves, thereby improving RWC. The enhanced resilience of the mesophyll airspace is believed to facilitate this improvement, possibly due to the expansion of the intercellular spaces [49]. These results suggest that optimizing light conditions can significantly influence water retention and overall plant health.

### 3.3. Effect of the LED Spectra on the Enzyme Activity

The results of our study demonstrated that the ABA content was significantly higher in all three varieties when exposed to red and blue light than in the control and R&B light conditions. This finding is consistent with the research by Palma et al. [50], who reported increased ABA levels in plants grown under blue or red light compared with those grown under white light. Blue light enhances the expression of genes involved in ABA biosynthesis [51]. The lower ABA levels observed under the control conditions may be attributed to the absence of specific light wavelengths that promote ABA synthesis, indicating that the enhanced light spectrum provided by the red and blue LEDs plays a crucial role in the regulation of ABA.

Our study found that LED exposure had no significant effect on the antioxidant enzyme activity in kale and collard cultivars. In contrast, the cabbage cultivars exhibited reduced antioxidant enzyme activity in response to increased light exposure. However, we acknowledge the possibility that control plants grown under natural light conditions may have already been experiencing light stress, which could have influenced the observed enzyme activities. The lack of variation in enzyme activity between plants exposed to different levels of light and control plants indicates that this type of light does not induce light stress.

The increased production of ROS, particularly superoxide radicals that convert to hydrogen peroxide, may explain the elevated antioxidant enzyme activity observed in untreated and treated kale and collard greens [10]. Under adverse stress conditions, ROS are generated during fundamental metabolic processes in various subcellular compartments, including chloroplasts, during photosynthesis. Excessive ROS can damage plant tissues; thus, plants rely on antioxidant systems, including antioxidant enzymes, to mitigate this damage [52]. In a study by Li et al. [47], Chinese cabbage treated with R&B light exhibited a higher O_2_^−^ generation rate compared to the control, triggering a stress response that resulted in increased SOD and CAT activities. Additionally, Zhou et al. [53] reported that LED irradiation reduced the MDA content while maintaining the activity of various antioxidant enzymes in pak choi, highlighting the potential of optimized light treatments to enhance plant resilience.

### 3.4. Effect of LED Spectra on the GSLs Content

GSLs, a diverse group of over 130 hydrophilic, sulfur-containing secondary metabolites, are predominantly found in *Brassica* crops and seeds [4]. The composition of aliphatic and indolyl GSLs is influenced by various factors, including genotype, environmental conditions, and genotype-environment interactions [54]. Notably, light conditions can significantly enhance GSL levels; for instance, broccoli sprouts exposed to light exhibit higher GSL content than those grown in darkness [55]. Similarly, increased light intensity has been shown to elevate GSL concentrations in pak-choi leaves and broccoli seedlings compared with those cultivated under low light conditions [56].

Zhuang et al. [57] demonstrated that exposure to yellow, green, blue, and purple LED lights resulted in higher glucoraphanin and glucoerucin contents in broccoli, attributed to the upregulation of genes involved in aliphatic GSL biosynthesis under these light conditions. In particular, blue light significantly increased glucoraphanin and total GSLs levels compared with white light. Conversely, in cabbage microgreens, the combination of red and blue (R+B) light enhanced aliphatic GSL levels, whereas red light alone reduced their levels [29]. Xue et al. [58] also reported that an increased ratio of blue light positively influenced the accumulation of aliphatic GSLs in broccoli sprouts, which was associated with the upregulation of key biosynthetic genes. The effects of light spectra on GSLs production vary not only among different Brassica species but also within specific cultivars, likely due to genetic and morphological differences. For instance, progoitrin and gluconapin levels in cabbage were lower under R&B light than under other light treatments. In contrast, kale exhibited lower levels of these GSLs under control and red light conditions. Interestingly, the levels of glucobrassicin and gluconasturtiin were elevated under red light compared with the control. These differential responses could be attributed to the unique genetic backgrounds and phytochemical profiles of the cultivars, which may influence the expression and regulation of the genes involved in GSL biosynthesis pathways in response to different light spectra. These findings highlight the complexity of GSLs regulation in response to light and underscore the importance of optimizing light conditions to enhance the nutritional quality of *B. sativa*.

### 3.5. Effect of the LED Spectra on the Mineral Element Content

Therefore, a high potassium content may enhance the growth of the red-blue, blue, and red LED treatments. Potassium influences plant growth [59]. Increased potassium levels in plants decrease ROS production [60]. Potassium maintains the photosynthetic electron transport activity while decreasing the activity of nicotinamide adenine dinucleotide phosphate (NADPH) oxidases, thereby lowering ROS [61].

Red or a mixture of red and blue (red: blue = 2) significantly increased the accumulation of iron, phosphorus, magnesium, and nitrogen in the lettuce tissues, whereas blue significantly increased the accumulation of potassium and calcium [62]. However, there were no differences in nitrogen accumulation between blue and red monochromatic light or different red-to-blue light ratios among lettuce leaves [63]. A red: blue ratio of three increased lettuce growth capacity, nutrient uptake (nitrogen, phosphorus, potassium and magnesium), and energy and land surface utilization efficiency [64].

Red light has been shown to efficiently reduce nitrate concentrations in plants by stimulating nitrate reductase activity through phytochromes [65]. Consistent with these findings, the present study demonstrated that red light decreased urea levels in cabbage by increasing nitrate reductase activity. However, we recognize that in kale leaves under R&B light conditions, a significant increase in nitrate reductase activity did not lead to a corresponding change in urea content. This discrepancy suggests that other regulatory mechanisms may be involved in kale production. Similarly, Brazaitytė et al. [66] reported a reduction in nitrates in kale microgreens under monochromatic red light. Nitrate reductase is a rate-limiting enzyme that catalyzes the conversion of nitrate into nitrite in the cytoplasm. The resulting nitrite is then converted to ammonia in chloroplasts under light conditions by nitrite reductase [67]. Red and blue light play crucial roles in the regulation of nitrate reductase activity and gene expression [68]. According to Fan et al. [69], the low nitrate and nitrite concentrations in pak-choi under red and blue light are due to the higher activity of nitrate and nitrite reductases. This indicates the potential post-translational regulation of these enzymes by red and blue light via different light receptors, which may vary among plant species [70].

## 4. Materials and Methods

### 4.1. Plant Materials and Experimental Design

Three cabbage cultivars of *B. oleracea* were selected for this study based on their significant agricultural importance and consumer demand in the local market. These cultivars were chosen because they are among the most widely cultivated Brassica varieties in our region. The *B. oleracea* var. acephala (kale) seeds were obtained from the Seed-Saver Exchange Company. This open-pollinated variety has been maintained and selected for its robust growth and cold-tolerance characteristics. The *B. oleracea* var. viridis (collard) seeds were purchased from Nazboo. This commercial F1 hybrid variety is known for its large, tender leaves and resistance to bolting. The *B. oleracea* var. capitata (cabbage) seeds were also obtained from Nazboo. This open-pollinated cabbage variety was selected for its uniform head formation and storage capabilities. These cultivars exhibit distinct morphological and agronomic traits that contribute to their adaptability and yield potential under specific environmental conditions in the region.

The experiment followed a factorial design within a randomized setup with three replicates at the Isfahan University of Technology in April 2016. The number of samples corresponds to the number of replicates. The entire experiment was repeated using at least three biological repeats. Seeds were sown in growing media of cocopeat, perlite, and peat moss in a 2:1:1 ratio in a greenhouse located at approximately 32.6542° N latitude and 51.6728° E longitude. The greenhouse environment was maintained at 18 °C at night and 25 °C during the day with relative humidity between 60% and 85%. Healthy and uniform seedlings were transferred to 5 kg pots (height of 15 cm, a diameter of 25 cm, with a soil volume of 6000 cm^3^) filled with a mixture of soil, sand (1:1 *w*/*w*) with pH = 7.5, EC = 2.8 dS m^−1^, and 1.92% organic matter. During the first week of the experiment, the plants received a half-strength nutrient solution, transitioning to a complete nutrient solution thereafter, based on Jones’ formula. This included the following nutrient concentrations: 116 mg L^−1^ N, 21 mg L^−1^ P, 82 mg L^−1^ K, 125 mg L^−1^ Ca, 21 mg L^−1^ Mg, 28 mg L^−1^ S, 6.8 mg L^−1^ Fe, 1.97 mg L^−1^ Mn, 0.25 mg L^−1^ Zn, 0.70 mg L^−1^ B, 0.07 mg L^−1^ Cu, and 0.05 mg L^−1^ Mo, provided by KH_2_PO_4_, NH_4_NO_3_, Ca (NO_3_)_2_, CuSO_4_, Fe EDTA, MnSO_4_, ZnSO_4_, Na_2_MoO_4_, and H_3_BO_3_ [71].

### 4.2. Lighting Control System for LED Applications

Polyurethane foam sheets of 3 cm thickness insulated the 1.5 × 0.6 × 1 m growth room. Heat transfer from the warm copper block to the cold copper block through the chamber walls, floor, and ceiling may provide a linear temperature differential between 26 °C, 18 °C, and 25 °C (each 1 cm thick). The temperature was measured using a digital thermometer (ATE040, Arvin Tajhiz Espadana Co., Tehran, Iran) with a resolution of 0.1 °C.

The experimental setup consisted of four growth cabinets, each equipped with three control units to manage 120 LED lights. The LED arrays were sourced from OSRAM (Munich, Germany) and emitted light in the red (650–665 nm), blue (460–465 nm), and a red-blue (70:30 ratio) spectrum.

All LED lights had a power rating of 1.0 W and were driven by a 2 A power source providing 110 VDC. This setup ensured a consistent photosynthetic photon flux density (PPFD) of 100 ± 15 μmol m^−2^ s^−1^ across the different LED light spectra. For the natural light control treatment, the plants were grown in a greenhouse, where the PPFD was measured to range from 200 to 320 μmol m^−2^ s^−1^, with a mean of 260 ± 15 μmol m^−2^ s^−1^. This provided an environment similar to the LED-lit growth chambers, with the only significant difference being the light source.

The spectral properties of the LED arrays were characterized based on the well-established emission profiles of horticultural red and blue LEDs. Blue LEDs (InGaN-based) and red LEDs (AlGaInP-based) emit in narrow and highly reproducible wavelength bands determined by their semiconductor bandgap energies. In accordance with the standard spectral output of LEDs in these regions, the blue diodes used in the lighting system exhibit a peak wavelength of approximately 460–465 nm with a full width at half maximum (FWHM) of about 20–25 nm, while the red diodes emit with a peak at roughly 650–665 nm and a similar bandwidth of 18–25 nm. For the red–blue arrays, the overall spectral distribution was obtained by combining the individual red and blue emission bands in proportion to the 70:30 diode ratio. These spectral characteristics accurately represent the expected output of horticultural LEDs operating within these wavelength ranges and provide a reliable description of the light environment used in the experiment.

To maintain the desired light intensity at the leaf surface, a potentiometer was used to control the light levels up to a maximum of 500 μmol m^−2^ s^−1^. During the experiments, the light intensity was monitored using a LI-250A light meter and a LI-190 two-quantum sensor (LI-COR Inc., Lincoln, NE, USA).

In addition, a 0.72 K (50 W) power resistor was included as a current limiter, and input/output capacitors were added to enhance the transient responsiveness of the system. This setup was replicated in each of the four growth cabinets, and each LED array was equipped with a heat sink to ensure adequate cooling.

A microcontroller with an ASM51 assembly language-based logic control system (Arvin Tajhiz Espadana Co.,Tehran, Iran) [72] was integrated into each growth cabinet to regulate the environmental conditions, including temperature, LED brightness, and the 16/8 h light/dark photoperiod.

### 4.3. Harvesting and Growth Parameters

Upon reaching maturity, the plant materials were harvested, and the age of the plants at harvest time was based on market standards and consumer preferences. Specifically, kale and collard were harvested 60 days after planting, and cabbage was harvested 70 days after planting. The number of leaves, plant height, and fresh weight was recorded using a ruler and balance. The leaf width was measured using a digital caliper in mm. Each sample was immediately placed in liquid nitrogen for subsequent analysis. Fresh and dry weights were determined at harvest using a precise 0.01 g balance, with dry weights recorded after drying the samples at 70 °C. The root volume of the plants was measured using a water displacement method in mL. The leaf count per plant, shoot length, leaf length, and petiole length were also documented [73].

### 4.4. Chlorophyll Index

The SPAD levels of the leaves were assessed using a SPAD-502 Plus chlorophyll meter (Osaka, Japan). Three measurements were taken from three different leaves of each plant (nine measurements per replication), and the average value was reported.

### 4.5. Chlorophyll Fluorescence

Chlorophyll fluorescence was assessed using a chlorophyll fluorometer (RS232 Handy PEA, King’s Lynn, Norfolk, UK) from 8:00 to 9:00 a.m. After a 30 min dark acclimation, both maximum (Fm) and variable (Fv) fluorescence were recorded. In addition, the maximum photochemical efficiency of photosystem II (Fv/Fm) was calculated [74].

### 4.6. Relative Water Content (RWC)

The relative water content was measured using 7 mm leaf disks. The fresh weight (FW) of the disks was determined for each treatment. The disks were hydrated for 48 h at 5 °C in the dark to reach a constant weight (TW), followed by oven-drying for 24 h at 105 °C to determine the dry weight (DW). The RWC was calculated using the following formula: RWC% = (FW − DW)/(TW − DW) × 100 [75].

### 4.7. ABA Content

One gram of fresh leaves was mixed with 10 mL of 80% methanol and homogenized with 0.1 g of polyvinylpyrrolidone at 4 °C. The sample was centrifuged for 15 min at 4000× *g*, and the supernatant was collected after adjusting the pH to 8. Following methanol evaporation, 5 mL of deionized water was added twice, and the mixture was treated with ethyl acetate before re-evaporation. The residue was then dissolved in 1 mL of a 3% methanol solution containing 0.1 M acetic acid. ABA was injected into a reverse-phase column (Diamonsic C18 5 m, 25 cm × 4.6 mm) using a 0.45 mm filter. A gradient of methanol-acetic acid (3–97%) was employed at a flow rate of 4 mL min^−1^ using a diode-array detector. Sigma-Aldrich standards (99.97% purity) were used for calibration based on the area under the curve. The ABA content was calculated using ng g^−1^ Fw [76].

### 4.8. Antioxidant Enzyme Activity

Following the Aebi method [77], catalase (CAT) activity was measured by monitoring the reduction in H_2_O_2_ absorbance at 240 nm using a spectrophotometer (Shimadzu UV160A, Kyoto, Japan). One unit of CAT activity was defined as the amount of enzyme needed to decompose 1.0 µM of H_2_O_2_ per minute [78]. Peroxidase (POX) activity was assessed using guaiacol as a substrate, and the increase in absorbance at 470 nm due to guaiacol oxidation was recorded over 3 min. One unit of POX activity was defined as the enzyme quantity required to oxidize 1.0 µM of guaiacol per minute [79]. Ascorbate peroxidase (APX) activity was determined by monitoring the rate of ascorbate oxidation at 290 nm with an extinction coefficient of 2.8 mM^−1^ cm^−1^. The protein content for calculating APX activity was determined using the Bradford method, with bovine serum albumin as the standard. One unit of APX activity was defined as the amount of enzyme required to oxidize 1 µM of ascorbic acid (AsA) per mg of protein per minute [78].

The total superoxide dismutase (SOD) activity was assessed by measuring the inhibition of nitroblue tetrazolium (NBT) photochemical reduction [76]. One unit of SOD activity was defined as the amount of enzyme needed to inhibit NBT reduction by 50%, as detected spectrophotometrically at 560 nm using a Shimadzu UV160A (Kyoto, Japan). SOD activity is reported as units per mg of protein [78].

### 4.9. GSLs Content

To measure the GSLs content, fully developed leaves were treated at 120 °C for 2 h to inactivate myrosinase. The leaves were then ground, mixed with methanol/water solution, and filtered before being analyzed using an HPLC system. The mobile phase consisted of a formic acid and acetonitrile/water gradient. The eluate absorbance was monitored at 229 nm. The GSLs content was calculated based on µM g^−1^ Dw [80].

### 4.10. Concentrations of Nitrogen and Potassium

The Kjeldahl Digestion Apparatus 143 (V40, Tehran, Iran) was used to determine the nitrogen content [81]. The leaves harvested for nutrient analysis were washed with deionized water and oven-dried at 65 °C for 24 h. Subsequently, 0.5 g of the powdered samples was digested on an open hot plate at room temperature for 12 h using 7.5 mL of 65% HNO_3_ and 2.5 mL of 36% HCl until a lightly colored solution was obtained. The remaining substance was purified and transferred to a 50 mL container using purified water and Whatman 4 filter paper. Nitrogen levels were analyzed using an automated Kjeldahl apparatus (V40, Iran). This was followed by heating the mixture at 105 °C for 2 h. The potassium was identified through flame spectroscopy (Model PFP7, Jenway, Stone, Staffordshire, UK) in triplicate [82,83]. The nitrogen and potassium contents were calculated based on mg kg Dw^−1^. To measure the urea content, leaf extraction was performed using boiling water. A mixture was prepared by combining 12 mL of H_2_SO_4_, 0.9 mL of diacetyl monoxime, 0.15 mL of thiosemicarbazide, and 0.15 mL of Fe_2_(SO_4_)_3_ with 10 mL of leaf extract. The mixture was then heated in a water bath at 85 °C for 30 min and subsequently cooled on ice until room temperature. The absorbance of the mixture was recorded at 520 nm using a spectrophotometer (Shimadzu UV160A-Kyoto, Japan). A stock standard solution of urea (µmol g^−1^ Dw) was created by dissolving a urea standard (Sigma-Aldrich, St. Louis, MO, USA) in distilled water, following the method outlined by Chen et al. [84]. The urea content was calculated using 100 mmol L^−1^.

### 4.11. Nitrate Reductase

The nitrate reductase activity of the leaf samples was measured using a phosphate solution (100 mg, pH = 7.5) containing 4% propanol and potassium nitrate and stored in the dark for 1 h at 30 °C. The absorbance at 540 nm was measured after adding sulfanilic acid, chloride acid, and naphthylethylene diamide solution, and the absorbance at 540 nm was measured. The enzymatic activity was calculated using mg NO_3_ g^−1^ Dw [85].

### 4.12. Statistical Analysis

A factorial experiment based on a randomized complete design with three replicates was performed. Two-way ANOVA was performed using Statistix 8 (Tallahassee, FL, USA), and the means were compared for significance using the least significant difference (LSD) test at *p* < 0.05. The principal component analysis (PCA) was carried out using Statgraphics Centurion Version XVI.

## 5. Conclusions

The findings of this study demonstrate that LED lights can effectively substitute for natural light, with different artificial light sources having varying impacts on the cultivars of leafy cabbage examined. For the three leafy cabbage cultivars kale (*Brassica oleracea.* var. acephala), collard (*Brassica oleracea* var. viridis), and cabbage (*Brassica oleracea* var. capitata), the use of red-blue (R&B) LED lights was the most effective in increasing various growth-related parameters, such as shoot and root fresh/dry weight, shoot length, leaf length and width, and number of leaves. This suggests that the R&B light spectrum provides the optimal conditions for enhancing the overall growth and development of these leafy Brassica crops. Interestingly, the application of blue light specifically resulted in a significantly higher glucosinolate (GSL) content in the cabbage cultivar, which may enhance the nutritional value and health benefits of this vegetable. The metabolism and phytochemical properties of the leafy cabbage cultivars were influenced by both the cultivar type and light quality. Additionally, the increased potassium and nitrogen levels observed under LED lighting, particularly the R&B spectrum, had a favorable impact on the growth of various parameters and chlorophyll content across the three cultivars. This suggests that the quality of light not only affects the phytochemical composition but also plays a crucial role in plant nutrient uptake and utilization. Overall, these results provide novel insights into the effects of different LED light spectra on the growth, nutritional, and phytochemical properties of leafy cabbage cultivars. The red-blue light combination was identified as the most effective for promoting desirable traits, making it a recommended option for the optimal cultivation of these Brassica vegetables.

## Figures and Tables

**Table 1:** Effects of Various LED Light Wavelengths on the Growth Traits and Physiology of Selected Vegetables.

Type of LED Illumination	Control Conditions	Parameter Changes	Vegetable	Reference
Red: Blue	-	Total GSLs, polyphenols and flavonoids contents, antioxidant activities ▲	Chinese Cabbage and Kale	[2]
Red + Green + Blue	White light	Transpiration, stomatal conductance, and intracellular CO_2_ concentration ▲	Chinese cabbage	[12]
Red: Blue ratio = 3	Fluorescent light	Leaf antioxidant capacity, concentrations of caftaric and chicoric acids, nutritional properties ▲	Lettuce	[13]
Red: Blue	Blue or red	Anthocyanin, total phenolic, total flavonoid, ascorbic acid contents ▲	Broccoli sprouts	[14]
Red: Blue ratio = 7:2	No light supplementation	Photosynthetic enzymes, photosynthetic pigments, photosynthetic capacity ▲	Tomato	[15]
Red: Blue ratio = 6:3	White LED light	Dry matter ▼ Soluble proteins, total glucosinolate, total anthocyanin, flavonoid, antioxidant activity ▲	Chinese kale	[16]
Red: Blue ratio = 1:2	Red: Blue = 1:1	Contents of carotenoid, soluble protein, soluble sugar, vitamin C, total flavonoids, total polyphenol and contents of total glucosinolates ▲	Broccoli seedling	[17]

LED: Light Emitting Diode. ▲ or ▼, enhanced or decreased compared with the control.

## Data Availability

Data are contained within the article and Supplementary Materials.

## Supplementary Materials

The following supporting information can be downloaded at: https://www.mdpi.com/article/10.3390/plants14233700/s1. Table S1: Analysis of variance of variety and light on some growth characteristics of three leafy cabbage; Table S2: The main effect of variety and light on some growth characteristics of three leafy cabbage; Table S3: Analysis of variance of variety and light on some growth characteristics of three leafy cabbage; Table S4: The main effect of variety and light on some growth characteristics of three leafy cabbage; Table S5: Analysis of variance of variety and light on ABA, some antioxidant enzymes and elements of three leafy cabbage; Table S6: The main effect of variety and light on ABA, some antioxidant enzymes of three leafy cabbage; Table S7: The main effect of variety and light on some elements of three leafy cabbage; Table S8: Analysis of variance of variety and light on glucosinolate of three leafy cabbage; Table S9: The main effect of variety and light on glucosinolate of three leafy cabbage.
