# Supplementary material for: The Nutritional Value, Biochemical Traits, and Growth of Brassica oleracea Grown Under Red, Blue, and Combined Red–Blue LED Lighting
Source: Plants (Basel). 2025 Dec 4;14(23):3700. doi: 10.3390/plants14233700 (PMC12694474; doi:10.3390/plants14233700)
Supplement: Supplementary file 1 [file plants-14-03700-s001.zip › plants-3980314-supplementary.pdf]

Table S1. Analysis of variance of variety and light on some growth characteristics of three leafy cabbage.

Treatments	df	Shoot fresh weight	Shoot dry weight	Root fresh weight	Root dry weight	Chlorophyll index	Chlorophyll fluorescence	Root volume	Relative water content
Variety	2	0.40 ^{ns}	0.00 ^{ns}	0.00 ^{ns}	0.00^{ns}	53.49 ^{ns}	0.00**	0.12 ^{ns}	1141.08**
Light	3	1.74**	0.01**	0.00**	0.00 ^{ns}	424.89**	0.00**	2.85**	179.94**
Variety× Light	6	0.05**	0.00**	0.00**	0.00**	51.56**	0.00**	0.28**	233.88**
Error	24	0.16	0.00	0.00	0.00	38.73	0.00	0.30	56.81
CV		51.74	42.35	60.98	18.88	27.08	3.08	52.92	8..12

ns: no significant, ** significant at 1% and * significant at 5%. The confidence interval was calculated with a confidence level of 95%.

Table S2. The main effect of variety and light on some growth characteristics of three leafy cabbage.

Treatments	Shoot fresh weight (g)	Shoot dry weight (g)	Root fresh weight (g)	Root dry weight (g)	Chlorophyll index (SPAD value)	Chlorophyll fluorescence (Fv/Fm)	Root volume (mL)	Relative water content (%)
Variety								
Kale	0.99a	0.06a	0.04a	0.05a	23.37a	0.79b	0.94a	104.09a
Collard	0.70a	0.07a	0.06a	0.03a	20.70a	0.81ab	1.15a	87.05b
Cabbage	0.65a	0.05a	0.07a	0.03a	24.87a	0.82a	1.04a	87.35b
Light								
Control	0.38b	0.03b	0.03b	0.01a	20.98b	0.81a	0.69b	96.90a
Blue-Red	1.40a	0.11a	0.06ab	0.05a	33.27a	0.82a	1.88a	95.58a
Blue	0.60b	0.05b	0.03b	0.07a	17.98b	0.78b	0.84b	91.93ab
Red	0.75b	0.05b	0.104a	0.02a	19.69b	0.82a	0.75b	86.91b

Within a column in each treatment means followed by the same letter are not significantly different at P<5% according to the least significant difference test.

Table S3. Analysis of variance of variety and light on some growth characteristics of three leafy cabbage.

	df	Petiole length	Shoot length	Leaf width	Number of leaves per plant	Leaf length
Variety	2	47.16**	52.76**	2.19**	36.63**	0.91ns
Light	3	15.30**	45.61**	5.82**	18.66**	14.43**
Variety× Light	6	1.58**	28.74**	0.73**	1.47**	0.76**
Error	24	0.56	4.40	0.56	2.28	0.89
CV		14.97	19.07	19.71	22.07	19.57

ns: no significant, ** significant at 1% and * significant at 5%. The confidence interval was calculated with a confidence level of 95%.

Table S4. The main effect of variety and light on some growth characteristics of three leafy cabbage.

Treatments	Petiole length (cm)	Shoot length (cm)	Leaf width (mm)	Number of leaves (per plant)	Leaf Length (cm)
Variety					
Kale	7.23a	12.39a	3.287b	6.27b	4.78a
Collard	4.41b	9.44b	4.30a	8.70a	5.11a
Cabbage	3.40c	11.16ab	3.81ab	9.66a	4.57a
Light					
Control	3.40c	9.19b	3.04c	7.36bc	3.49c
Blue-Red	5.41b	12.22a	4.90a	9.88a	6.48a
Blue	6.52 a	11.03ab	3.39bc	6.71c	4.29bc
Red	4.72b	11.55a	3.87b	8.88ab	5.03b

Within a column in each treatment means followed by the same letter are not significantly different at P<5% according to the least significant difference test.

Table S5. Analysis of variance of variety and light on ABA, some antioxidant enzymes and elements of three leafy cabbage.

	df	ABA	APX	SOD	POX	CAT	K	Urea	Nitrogen	Nitrate reductase
Variety	2	36.59**	3.01**	8.64**	5.78**	0.36**	4.57**	0.29 ns	20.42**	0.04**
Light	3	949.94 ns	0.13**	0.34 ns	0.35**	0.00 ns	27.63**	0.06 ns	1.99**	0.03**
Variety× Light	6	137.50**	0.12**	0.31**	0.37**	0.01**	6.99**	1.47**	8.58**	0.08**
Error	24	0.04	0.05	0.16	0.04	0.01	0.81	0.43	0.43	0.00
CV		0.41	11.95	28.29	11.72	28.25	16.69	5.05	9.70	27.84

ns: no significant, ** significant at 1% and * significant at 5%. The confidence interval was calculated with a confidence level of 95%.

Table S6. The main effect of variety and light on ABA, some antioxidant enzymes of three leafy cabbage.

Treatments	ABA content (ng gr $^{-1}$ FW)	APX (unit mg^{-1} protein)	SOD (unit mg^{-1} protein)	POX (unit mg^{-1} protein)	CAT (unit mg^{-1} protein)
Variety					
Kale	47.58c	2.16a	1.93a	2.20a	0.46a
Collard	50.32b	2.17a	1.92a	2.21a	0.54a
Cabbage	50.83a	1.30b	0.45b	1.00b	0.21b
Light					
Control	39.46d	1.73b	1.34a	1.82 b	0.43a
Blue-Red	42.06c	1.81ab	1.33 a	1.69b	0.39a
Blue	57.37b	1.96a	1.34 a	1.64b	0.37a
Red	59.43a	2.00a	1.73a	2.08a	0.44a

Within a column in each treatment means followed by the same letter are not significantly different at P<5% according to the least significant difference test.

Table S7. The main effect of variety and light on some elements of three leafy cabbage.

Treatments	K (mg kg^{-1} Dw)	Urea (µmol g^{-1} Dw)	Nitrogen (mg kg^{-1} Dw)	Nitrate reductase (mg NO_3 g^{-1} Dw)
Variety				
Kale	5.49ab	12.85a	5.83b	0.23a
Collard	4.77b	13.08a	6.28b	0.10c
Cabbage	5.99a	13.15a	8.28a	0.17b
Light				
Control	2.85b	13.34a	6.11b	0.13b
Blue-Red	6.45a	13.13a	7.09a	0.23a
Blue	6.61a	12.88a	7.08a	0.10b
Red	5.76a	12.76a	6.93a	0.21a

Within a column in each treatment means followed by the same letter are not significantly different at P<5% according to the least significant difference test.

Table S8. Analysis of variance of variety and light on glucosinolate of three leafy cabbage.

	df	Glucoraphanin	Progoitrin	Gluconapin	Glucobrassicin	Gluconasturtiin
Variety	2	72.27**	39.26**	115.50**	277.39**	1.84**
Light	3	13.73**	1.75**	16.75**	61.88**	0.27**
Variety× Light	6	11.29**	5.21**	12.00**	46.24**	0.15**
Error	24	0.55	0.56	1.50	1.74	0.03
CV		4.44	9.21	8.75	4.70	8.95

ns: no significant, ** significant at 1% and * significant at 5%. The confidence interval was calculated with a confidence level of 95%.

Table S9. The main effect of variety and light on glucosinolate of three leafy cabbage.

Treatments	Glucoraphanin (µM g^{-1} DW)	Progoitrin (µM g^{-1} DW)	Gluconapin (µM g^{-1} DW)	Glucobrassicin (µM g^{-1} DW)	Gluconasturtiin (µM g^{-1} DW)
Variety					
Kale	14.35c	6.36c	10.95c	23.43c	1.63c
Collard	16.66b	8.05b	13.97b	27.87b	2.13b
Cabbage	19.26a	9.97a	17.16a	33.04a	2.40a
Light					
Control	14.93b	7.50 b	12.30c	24.28c	1.79b
Blue-Red	17.09a	8.19ab	13.59b	28.61b	2.13a
Blue	17.47a	8.28a	14.94a	29.62ab	2.12a
Red	17.53a	8.54a	15.28a	29.95a	2.17a

Within a column in each treatment means followed by the same letter are not significantly different at P<5% according to the least significant difference test.
